# Plasma exosomal caveolin-1 predicts Poor Prognosis in Ovarian Cancer

**DOI:** 10.7150/jca.58762

**Published:** 2021-06-16

**Authors:** Lijuan Yang, Haohao Wu, Yan Zhu, Xiaoping Chen, Youguo Chen

**Affiliations:** 1 Department of Obstetrics and Gynecology, The First Affiliated Hospital of Soochow University, Suzhou, Jiangsu, 215006, P.R. China.; 2 Department of Obstetrics and Gynecology, The First People's Hospital of Yancheng, Yancheng, Jiangsu, 224001, P.R. China.; 3 Department of Radiotherapy, The First People's Hospital of Yancheng, Yancheng, Jiangsu, 224001, P.R. China.

**Keywords:** caveolin-1, ovarian cancer, exosomal, prognosis, biomarker

## Abstract

**Objective:** In this study, we aimed to evaluate the levels of plasma exosomal caveolin-1(CAV1) and determine its prognostic value in ovarian cancer patients.

**Patients and Methods:** Exosome-rich fractions were isolated from the plasma of 155 patients with ovarian cancer. TEM, NTA and western blot analysis were used to confirm the exosome integrity and purification.

**Results:** Compared with healthy controls, plasma exosomal CAV1 levels in ovarian cancer patient were significantly down-regulated (*P* < 0.001). The low plasma levels of exosomal CAV1 in ovarian cancer patient plasma were related to FIGO stages, grades and lymph node metastasis (all *P* < 0.01). Among all ovarian cancer patients, DFS was worse in patients who had low plasma exosomal CAV1 levels compared with that in patients with high plasma exosomal CAV1 levels (*P* < 0.001). The OS of patients with low plasma exosomal CAV1 levels was shorter than that in patients with high plasma exosomal CAV1 levels (*P* < 0.001). The AUROC of plasma exosomal CAV1 was 0.76 (95% CI: 0.68-0.82) for DFS prediction in ovarian cancer patients, with a sensitivity 52.9 (95% CI: 42.8-62.9) and a specificity 88.7 (95% CI: 77.0-95.7). For OS prediction in ovarian cancer patients, the AUROC of plasma exosomal CAV1 was 0.78 (95% CI: 0.70-0.84), with a sensitivity 65.1 (95% CI: 49.1-79.0) and a specificity 81.2 (95% CI: 72.8-88.0).

**Conclusions:** Low exosomal CAV1 levels were closely related to the FIGO stages I/II, low grade, lymph node metastasis and prognosis of ovarian cancer patients. Plasma exosomal CAV1 may be a potential biomarker for the prognosis in ovarian cancer patients.

## Introduction

Ovarian cancer has the worst prognosis among the three major malignant tumors of the female reproductive system 1,2. Its incidence ranks third among gynecological tumors in China, and its case fatality rate ranks first, which pose a serious threat to the lives of female patients 3. There are 240,000 new cases of ovarian cancer and 150,000 deaths each year globally, making it the most malignant tumor among gynecological malignancies 4. The symptoms of ovarian cancer are insidious at the early stage. About 75% of ovarian cancer patients have developed to the late stage of the disease and are accompanied by extensive metastasis when they are explicitly diagnosed 5. And the cancer often cannot be completely removed by surgery. At present, the main treatment for ovarian cancer is still chemotherapy or radiotherapy. Following the development of biotechnology and genetic engineering, the study of differential gene expression during tumor pathogenesis can promote individualized treatment and drug delivery, making it possible to become the effective key target genes to diagnose and treat cancer 6,7. However, so far, there is still a lack of effective tumor markers for early diagnosis and prognosis evaluation of ovarian cancer patients.

Caveolae is a kind of invagination structure with flask-shaped cell membrane, which participates in many life activities of cells 8,9. Caveolae is the most important type of lipid rafts, composed of cholesterol, sphingolipids and protein, which is also the marker protein 10. As a key structural protein of caveolin, caveolin-1 (CAV1) is considered as a candidate tumor suppressor gene 11,12. In recent years, the role of CAV1 in tumors has attracted more and more attention. Sanna et al. 13 found that CAV1 exerts a functional activity mediated, by directly binding to sequences of genes involved in proliferation in ovarian carcinoma cells. Zeng 14 and other studies found the correlation between the expression of CAV1 and clinicopathological parameters of ovarian cancer and its relationship with the prognosis.

In recent years, more and more studies have found that exosomes can play an important role in the occurrence and development of ovarian cancer by regulating the biological behavior of ovarian cancer 15,16. Studies have shown that exosomes can be detected in the ascites of about 85.4% ovarian cancer patients 17. These exosomes can induce dendritic cell (DC) precursor cells, DC and DC under the condition of coexistence of dendritic cells. The apoptosis of peripheral blood mononuclear cells ultimately inhibits the killing function of peripheral blood mononuclear cells. Skryabin et al. 18 have confirmed that caveolin-1 involved in the organizing of lipid rafts can be found in exosomes. However, there are few reports on the diagnostic and prognostic value of exosomes in patients with ovarian cancer. Therefore, our goal in this study is to evaluate the levels of plasma exosomal CAV1 and determine its prognostic value in patients with ovarian cancer.

## Materials and Methods

### Patients & clinical samples

We collected blood samples of 155 patients with ovarian cancer from the First People's Hospital of Yancheng, and the Second People's Hospital of Yancheng from February 2016 to March 2019. All the enrolled 155 patients underwent surgical resection. The inclusion criteria for patients with ovarian cancer were as follows: (1) all cases were initial; (2) ovarian cancer was diagnosed clinically and pathologically; (3) no other systemic diseases such as hypertension, diabetes and other endocrine diseases, hepatitis and other infectious diseases or other tumors; (4) patients had no preoperative hormone therapy, radiotherapy or chemotherapy and complete clinical data were available. The exclusion criteria were as follows: (1) Patients who received preoperative hormone therapy and radiation and chemotherapy; (2) incomplete clinical data; and, (3) lost to follow up.

The baseline clinical data of 155 patients with ovarian cancer were collected from medical records including demographic features, tumor size, lymph node metastasis, Federation International of Gynecology and Obstetrics (FIGO) stages, and pathological differentiation. Patients were followed up through April, 2020, with a median follow-up duration of 39.5 months (range: 12.0-50.0 months). The survival data were collected from follow-up records, and disease-free survival (DFS) and overall survival (OS) were calculated. DFS was defined as the duration from resection to disease recurrence, disease progression, or death. OS was defined as the time interval from resection to death. The follow-up results of the 155 patients enrolled in this study were obtained through medical records or telephone interviews.

Blood samples were collected from 50 healthy controls in the First People's Hospital of Yancheng, during the same period, aged 53-77 years, with a median age of 68 years. All specimens were collected after obtaining informed consent from the patients. The study was approved by the Ethics Committees of the First People's Hospital of Yancheng (identification nos. HMU [Ethics] 2020-K047).

### Plasma exosome isolation

Remove the cells 1- 2ml human plasma (1×PBS should be diluted 5 times) with centrifuged at 500×g at 4 °C for 5 min. The supernatant, 2000×g, centrifuged at 4 °C for 10 min to remove cell debris. Large vesicles after centrifuged at 10 000×g at 4 °C for 30 min were removed. The large particles which may be mixed in the operation process was removed by filtering the supernatant with a 0.45 μm filter. The filtered supernatant was taken to the ultracentrifugation tube and 1 × PBS buffer solution was added to fill up the remaining volume to weigh and balance accurately. Put the tube on the rotor of the ultracentrifuge, centrifuge at 100000×g, 4 °C for 2 hours. The supernatant was discarded after centrifugation and at the bottom of the tube translucent sediment could be seen. The sediment was resuspended in 1×PBS buffer and centrifuged at 100 000×g at 4 °C for 80 min. 100-200 μl 1×PBS buffer was used to resuspend the exosomes which was transferred to 1.5 ml EP tube. Downstream experiments can be carried out directly and stored at -80 °C according to the requirements of follow-up experiments.

### Transmission electron microscopy (TEM)

Prepare the extracted exosome from the -80 °C refrigerator and thaw. Take 10 μl of the thawed exosome and add it dropwise to the front surface of the clean copper mesh slowly whose diameter is 2 mm. After the liquid completely infiltrates the entire mesh surface, wiped off the residual liquid and let it stand at room temperature for 1-2 min. Pipet 20 μl of 2% phosphotungstic acid and drop it on the copper net, then dye exosome for 2 min at room temperature and wipe off the remaining liquid and dry. Continue to drip PBS buffer solution, rinse the copper mesh 3 times repeatedly, and use an incandescent lamp to dry it. Observe the shape of exosome under the electron microscope, take pictures and record.

### Nanoparticle tracking analysis (NTA)

Take 500 μl exosome after thawing and put it into Nano Sight NS300 Instrument. Set the parameters for detecting exosome particle size. Start testing by switching on the instrument, record experimental data, and use NTA 3.3 software for data analysis.

### Western blotting

Dilute the extracted exosomes with RIPA lysate by a certain multiple, then draw the diluted Exo 20 μl, add it to the test sample well of a 96-well plate, and place it at room temperature. For obtaining total proteins, exosomes were isolated and added to sodium dodecyl sulfate (SDS) buffer. By using SDS-PAGE gel total protein was separated and transferred onto PVDF (polyvinylidene difluoride) membranes (Millipore, USA). Membranes was blocked in 5% non-fat milk for 1 h and then incubated overnight at 4 °C with the indicated primary antibodies, including an Annexin V, TSG101, CD9 and CD63 were obtained from Santa Cruz Biotechnology, Inc., (Texas, USA). Finally, membranes were incubated by using secondary antibodies for 1 h at room temperature.

### ELISA

Residual cells were removed from the plasma sample and with 1 × PBS (1:500 dilution) cell fragments was diluted. On ice the exosomes were precipitated with 100 ml RIPA lysate for half an hour. PBS (1:3 dilution) was used to dilute the samples after shaking and mixing. Take out the ELISA plate coated with CAV1 antibody, add 1 well of blank control and 7 wells of gradient concentration standard respectively. The diluted exosome samples were 100 μl. After incubated at 37 °C for 60 min, discard the liquid in the well, and spin dry then add 100 μl of Solution A, cover the membrane, and bath in an oven at 37 °C for 60 minutes, and wash the plate 3 times. Add 100 μl solution B, cover with membrane, incubate in a 37 °C oven for 30 min, wash the plate 5 times. 90 μl of TMB substrate solution, cover with film, and develop color at 37 °C for 15 min in the dark. 50 μl of termination reaction solution. The microplate reader detects the absorbance value at 450 nm wavelength.

### Statistical analysis

Statistical analyses were performed with SPSS 24.0 software (IBM). If the continuous measurement is normally distributed, we present it as mean (SD); if it is not normally distributed, we present it as median (IQR). Chi square tests or Wilcoxon's rank-sum tests was used to perform the correlation analyses. Kaplan-Meier curves showed DFS and OS. Log-rank test was used to determine the differences in DFS and OS between groups. ROC curve analysis was used to assess the prognostic value of plasma exosomal CAV1 levels in ovarian cancer. Result with *P* value < 0.05 was deemed to consider significant.

## Results

### Baseline characteristics

The clinical characteristics of patients are shown in Table 1. Among the 155 enrolled ovarian cancer patients in this study, Eighty-one patients (52.26%) were less than 60 years of age, and seventy-four patients (47.74%) were 60 years of age or older. The pathological types included 102 cases (65.81%) of ovarian serous carcinoma, 36 cases (23.23%) of mucinous ovarian carcinom and 17 cases (10.96%) of ovarian endometrioid carcinoma. 23 (14.84%), and 132 patients (85.16%) had FIGO stages I/II, and III/IV disease, respectively. The numbers of patients with low grade (1/2), and high grade (3/4) were 34 (21.94%), and 121 (78.06%), respectively. Tumor size was less than 2 cm in 119 cases (76.77%) and greater than 2 cm in 36 cases (23.23%). Moreover, 59 patients (38.06%) had lymph node metastasis. In addition, the tumor position of 40 patients (25.81%), and 115 patients (74.19%) were in one side and bilateral, respectively.

### Characterization of exosomes isolated from plasma

**Figure 1:**
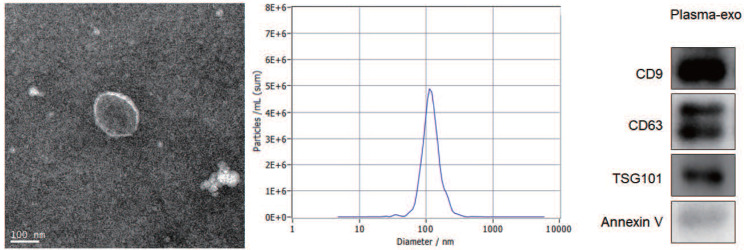
** Patient exosome characterization. (A)**TEM images showed that the exosomes were round or quasi circular vesicles with a diameter of about 40-100 nm, with complete capsule and clear background. **(B)**The NTA data revealed that the diameter of plasma exosomal CAV1 in ovarian cancer patients mainly concentrated in 60 -110 nm, and the maximum distribution peak was 102.5 nm. **(C)**Western blot analysis showed that the expression of exosome markers including Annexin V, Tsg101, CD9 and CD63 were found in plasma exosomes.

TEM, NTA and western blot analysis were used to confirm the exosome integrity and purification. The exosomes were obtained by gradient ultracentrifugation at low temperature and then fixed and stained. TEM images showed that the exosomes were clustered and connected with each other, with clear background. The diameter was between 100 nm and 200 nm. The shape was double disc like vesicle structure with intact lipid capsule (Figure 1A). The NTA data revealed that the median value of the total particles was about 100 nm, mainly distributed between 50 and 200nm, and the diameter of a small number of particles was between 0-50 nm (Figure 1B). Western blot showed that the expression of Annexin V, Tsg101, CD9 and CD63 were positive for plasma exosomes (Figure 1C).

### Correlation between plasma exosomal CAV1 levels and clinical pathological parameters

**Figure 2:**
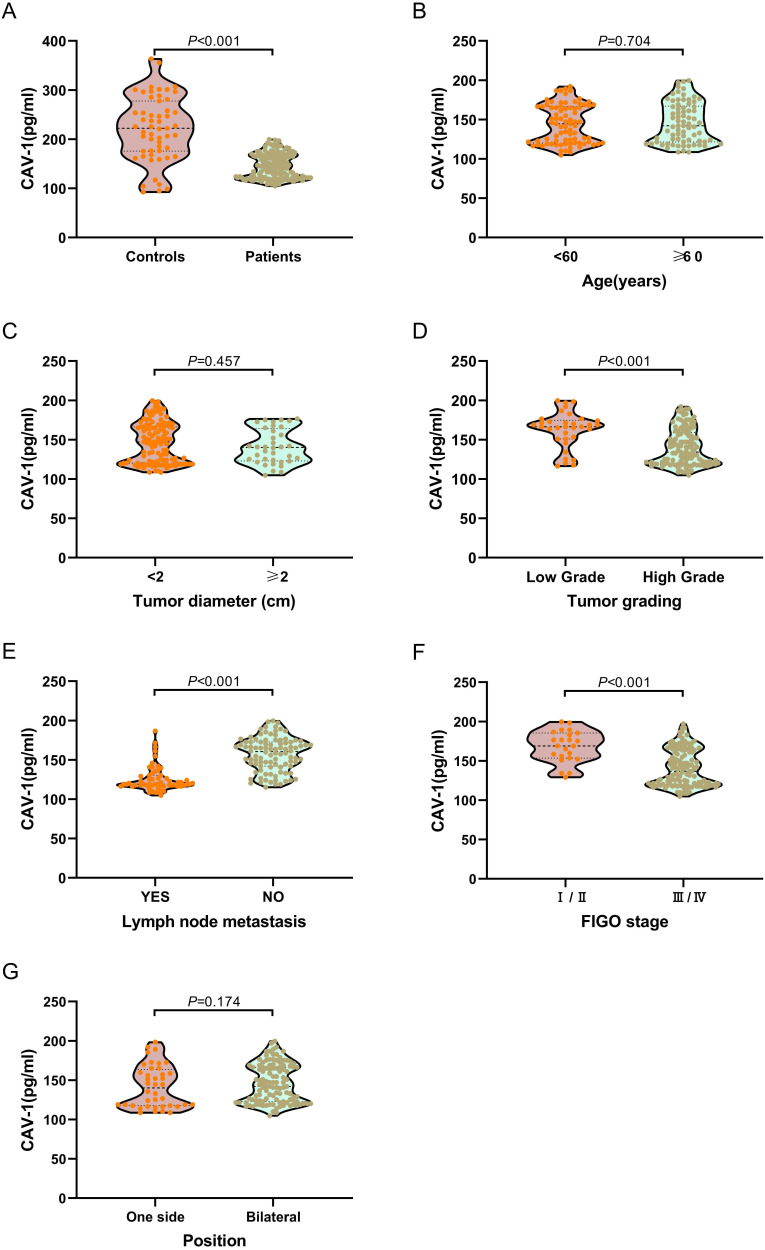
** Correlation of low plasma exosomal CAV1 levels with characteristics features in ovarian cancer patients. (A)** Compared with healthy controls, exosomal CAV1 levels in ovarian cancer patient plasma were significantly down-regulated (P < 0.001). **(B)** No significantly statistical difference of plasma exosomal CAV1 levels between different age (*P* = 0.704). **(C)** No significantly statistical difference of plasma exosomal CAV1 levels between tumor diameter (*P* = 0.457). **(D)** The levels of exosomal CAV1 in patient plasma were significantly higher in ovarian cancer patients with low grade (1/2) than high grade (3/4) (*P* < 0.001). **(E)** The levels of exosomal CAV1 in patient plasma were significantly higher in ovarian cancer patients with no lymph node metastasis than those with lymph node metastasis (*P* < 0.001). **(F)** The levels of exosomal CAV1 in patient plasma were significantly higher in ovarian cancer patients with FIGO stages I/II than FIGO stages III/ IV disease (*P* < 0.001); **(G)** No significantly statistical difference of plasma exosomal CAV1 levels between different tumor position (*P* = 0.174). **(H)** No significantly statistical difference of plasma exosomal CAV1 levels among different pathological types (*P* = 0.810).

We examined plasma exosomal CAV1 levels in ovarian cancer patients and healthy controls using ELISA. As shown in Figure 2A, exosomal CAV1 levels in ovarian cancer patient plasma were significantly down-regulated (*P* < 0.001), when compared with healthy controls. We also investigated plasma exosomal CAV1 levels in ovarian cancer patients at different disease stages. The levels of exosomal CAV1 in patient plasma were significantly higher in ovarian cancer patients with FIGO stages I/II, low grade (1/2) than FIGO stages III/IV disease and high grade (3/4) (both *P* < 0.01; Figure 2D, 2F). The levels of exosomal CAV1 in patient plasma were significantly higher in ovarian cancer patients with no lymph node metastasis than those with lymph node metastasis (*P* < 0.01; Figure 2E). However, there were no significantly statistical difference between patients with different age, tumor diameter, position and pathological types (All *P* > 0.05; Figure 2B, 2C, 2G, 2H).

**Figure 3:**
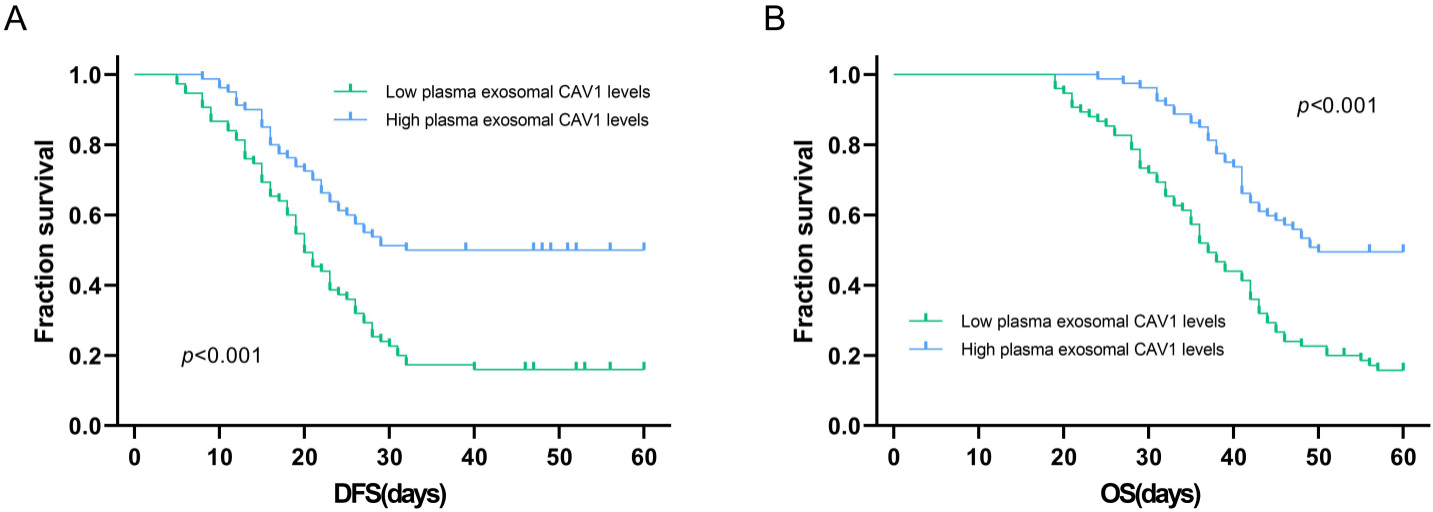
** Association of plasma exosomal CAV1 levels with DFS and OS in ovarian cancer patients. (A)** Among all ovarian cancer patients, DFS was worse in patients who had low plasma exosomal CAV1 levels compared with that in patients with high plasma exosomal CAV1 levels (*P* < 0.001). **(B)** Among all ovarian cancer patients, OS was worse in patients who had low plasma exosomal CAV1 levels compared with that in patients with high plasma exosomal CAV1 levels (*P* < 0.001).

Kaplan-Meier method was used to explore the relationship between plasma exosomal CAV1 levels and OS or DFS in ovarian cancer patients. Among all ovarian cancer patients, DFS was worse in patients who had low plasma exosomal CAV1 levels compared with that in patients with high plasma exosomal CAV1 levels (*P* < 0.001; Figure 3A). For OS, among all patients, the OS of patients with low plasma exosomal CAV1 levels was shorter than that of patients with high plasma exosomal CAV1 levels (*P* < 0.001; Figure 3B).

### Prognostic value of plasma exosomal CAV1 levels in ovarian cancer patients

We assessed the prognostic value of plasma exosomal CAV1 using ROC curve analysis (Table 2). The AUROC of plasma exosomal CAV1 was 0.76 (95% CI: 0.68-0.82) for DFS prediction in ovarian cancer patients. With the cutoff value of 130.56, the positive predictive value, positive likelihood ratio of plasma exosomal CAV1 were 90.0 (95% CI: 80.6-95.1), and 4.68 (95% CI: 2.2-10.2). The negative predictive value and negative likelihood ratio were 49.5 (95% CI: 43.8-55.1) and 0.53 (95% CI: 0.4-0.7) for prediction, with a sensitivity 52.9 (95% CI: 42.8-62.9) and a specificity 88.7 (95% CI: 77.0-9 5.7). For OS prediction in ovarian cancer patients, the AUROC of plasma exosomal CAV1 was 0.78 (95% CI: 0.70-0.84). With the cutoff value of 124.16, the positive predictive value, positive likelihood ratio of plasma exosomal CAV1 were 57.1 (95% CI: 46.1-67.5), and 3.47 (95% CI: 2.2-5.4). The negative predictive value and negative likelihood ratio were 85.8 (95% CI: 80.0-90.2) and 0.43 (95% CI: 0.3-0.7) for prediction, with a sensitivity 65.1 (95% CI: 49.1-79.0) and a specificity 81.2 (95% CI: 72.8-88.0).

## Discussion

Caveolae could provide a possible platform for cell signal transduction 19. It has been reported 20 that CAV1 expression is related to the clinical status of metabolic syndrome, and may become a potential target for the treatment and prevention of metabolic syndrome. CAV1 has also been confirmed to be expressed in lymphocytes and plays a potential role in latent HIV infection 21. In recent years, the role of *CAV1*, as a candidate tumor suppressor gene, has attracted increased attention 22,23. Many studies have shown that CAV1 is expressed in almost all normal cells, but the expression of CAV1 in most cancer cells or cells transformed by oncogenes is significantly reduced 24. CAV1 expression is associated with malignant transformation, proliferation, invasion, metastasis, signal transduction and multidrug resistance 25,26.

Vykoukal et al. 27 showed that CAV1 expression and secretion are associated with prostate cancer progression. An inverted CAV1 topology defines novel autophagy-dependent exosome secretion from prostate cancer cells. Ye et al. 28 showed that overexpression of CAV1 in patients with breast cancer administered neoadjuvant chemotherapy was associated with shorter DFS and OS. The high levels of CAV1 may serve as a prognostic biomarker for patients with breast cancer. In addition, circAKT1 acts as a sponge of miR-338-3p to facilitate clear cell renal cell carcinoma progression by upregulating CAV1 29. The study by Wang et al. 30 demonstrated that expression of CAV1 was positively associated with resistance of gastric cancer cells to cisplatin. Shi et al. 31 also showed the multifaceted roles of CAV1 on lung cancer occurrence, development and therapy.

In recent years, there has been an increase in studies on the expression and biological roles of CAV1 in ovarian cancer. Zeng et al. 14 showed that decreased expression of CAV1 mRNA in epithelial ovarian cancer (EOC) can predict poor overall survival. Expression of CAV1 protein in cancer cells is significantly associated with histological subtype of EOC, suggesting that CAV1 could serve as a useful prognostic biomarker and candidate therapeutic target in EOC. Sayhan et al. 32 demonstrated that expression of CAV1 in peritumoral stroma was associated with histological grade in ovarian serous tumors, suggesting that CAV1 acts as a differential diagnostic biomarker in ovarian serous tumors. Liu et al. 33 showed that miR-96-5p promoted the proliferation and migration of ovarian cancer cells by suppressing CAV1. However, the diagnostic and prognostic value of exosomes in patients with ovarian cancer remains unclear. To date, this is the first study to evaluate the levels of plasma exosomal CAV1 and determine its prognostic value in patients with ovarian cancer.

In this study, we extracted exosomes from the plasma of patients with ovarian cancer. TEM, NTA and western blot analysis were used to confirmed exosome integrity and purification. TEM images showed that the exosomes were clustered and connected with each other, with a clear background. The diameter was between 100 and 200 nm. The shape was a double disc-like vesicular structure with an intact lipid capsule. NTA data revealed that the median value of the total particles was about 100 nm, mainly distributed between 50 and 200 nm, and the diameter of a small number of particles was between 0 and 50 nm. Western blotting showed that expression of Annexin V, Tsg101, CD9 and CD63 was positive for plasma exosomes.

We examined plasma exosomal CAV1 levels in ovarian cancer patients and healthy controls using ELISA. Compared with healthy controls, exosomal CAV1 levels in ovarian cancer patient plasma were significantly downregulated. Next, we investigated plasma exosomal CAV1 levels in ovarian cancer patients at different disease stages. The levels of exosomal CAV1 in patient plasma were significantly higher in ovarian cancer patients with FIGO stages I/II, low grade (1/2) than in those with FIGO stages III/IV disease and high grade (3/4). The levels of exosomal CAV1 in patient plasma were significantly higher in ovarian cancer patients with no lymph node metastasis than in those with lymph node metastasis. However, there were no significant differences between patients with different age, and different tumor diameter, position and pathological type. Among all ovarian cancer patients, DFS was worse in patients who had low plasma exosomal CAV1 levels compared with patients with high plasma exosomal CAV1 levels. The OS of patients with low plasma exosomal CAV1 levels was shorter than that in patients with high plasma exosomal CAV1 levels.

Finally, we assessed the prognostic value of plasma exosomal CAV1 levels in ovarian cancer patients. The AUROC of plasma exosomal CAV1 were 0.76 (95% CI: 0.68-0.82) and 0.78 (95% CI: 0.70-0.84) for DFS and OS prediction in ovarian cancer patients, respectively. Plasma exosomal CAV1 levels has a better performance for OS and DFS prediction in ovarian cancer patients than CAV1 protein and/or mRNA and is a useful prognostic biomarker 14,34.

This study also had some limitations. First, although this was a large study evaluating plasma exosomal CAV1 levels in ovarian cancer patients, more patients from multiple centers need to be validated. Second, this study did not evaluate plasma exosomal CAV1 levels in the diagnosis of ovarian cancer. Finally, we did not explore the mechanism of action of exosomes and CAV1 in occurrence, development and therapy of ovarian cancer.

In summary, our study revealed that exosomal CAV1 levels in plasma of ovarian cancer patients were significantly downregulated. Low exosomal CAV1 levels were closely related to the FIGO stages I/II, low grade, lymph node metastasis and prognosis of ovarian cancer patients. These findings may facilitate the establishment of plasma exosomal CAV1 levels as a novel biomarker for prognosis in ovarian cancer.

## Figures and Tables

**Table 1:** Baseline characteristics of enrolled ovarian cancer patients

Characteristic	Ovarian cancer patients (n = 155)
**Age (years)**	
<60	81 (52.26)
≥60	74 (47.74)
**Tumor size (cm)**	
<2	119 (76.77)
≥2	36 (23.23)
**Tumor grading**	
Low Grade (1/2)	34 (21.94)
High Grade (3/4)	121 (78.06)
**Lymph node metastasis**	
YES	59 (38.06)
NO	96 (61.94)
**Pathologic type**	
Ovarian serous carcinoma	102 (65.81)
Mucinous ovarian carcinoma	36 (23.23)
Ovarian endometrioid carcinoma	17 (10.96)
**FIGO stage**	
I/II	23 (14.84)
III/ IV	132 (85.16)
**Position**	
One side	40 (25.81)
Bilateral	115 (74.19)

**Table 2:** The prognostic value of plasma exosomal CAV1 levels in ovarian cancer patients

Variable	Ovarian cancer patients (n = 155)
**AUROC (DFS)**	0.76 (0.68-0.82)
Cutoff value (95%CI)	130.56
Sensitivity, %	52.9 (42.8-62.9)
Specificity, %	88.7 (77.0-95.7)
Positive predictive value, %	90.0 (80.6-95.1)
Negative predictive value, %	49.5 (43.8-55.1)
Positive likelihood ratio	4.68 (2.2-10.2)
Negative likelihood ratio	0.53 (0.4-0.7)
**AUROC (OS)**	0.78 (0.70-0.84)
Cutoff value (95%CI)	124.16
Sensitivity, %	65.1 (49.1-79.0)
Specificity, %	81.2 (72.8-88.0)
Positive predictive value, %	57.1 (46.1-67.5)
Negative predictive value, %	85.8 (80.0-90.2)
Positive likelihood ratio	3.47 (2.2-5.4)
Negative likelihood ratio	0.43 (0.3-0.7)

## Abbreviations

CAV1: caveolin-1
DC: dendritic cell
FIGO: Federation International of Gynecology and Obstetrics
NTA: Nanoparticle tracking analysis
TEM: Transmission electron microscopy
DFS: disease-free survival
OS: overall survival

## References

[1] Peres LC, Bethea TN, Camacho TF (2020). Racial differences in population attributable risk for epithelial ovarian cancer in the OCWAA Consortium. J Natl Cancer Inst.

[2] da Costa AABA, Baiocchi G (2020). Genomic profiling of platinum-resistant ovarian cancer: The road into druggable targets. Semin Cancer Biol.

[3] Gao J, Han W, He Y (2020). Livin promotes tumor progression through YAP activation in ovarian cancer. Am J Cancer Res.

[4] Lin S, Yang H (2021). Ovarian cancer risk according to circulating zinc and copper concentrations: A meta-analysis and Mendelian randomization study. Clin Nutr.

[5] Wu J, Shang AQ, Lu WY (2016). Clinical significance of NGAL and MMP-9 protein expression in epithelial ovarian cancers. Int J Clin Exp Med.

[6] Zhang Q, Zhou W, Yu S (2021). Metabolic reprogramming of ovarian cancer involves ACSL1-mediated metastasis stimulation through upregulated protein myristoylation. Oncogene.

[7] Troisi R, Bjørge T, Gissler M (2018). The role of pregnancy, perinatal factors and hormones in maternal cancer risk: a review of the evidence. J Intern Med.

[8] Wang J, Bai Y, Zhao X (2018). oxLDL-mediated cellular senescence is associated with increased NADPH oxidase p47phox recruitment to caveolae. Biosci Rep.

[9] Dudãu M, Codrici E, Tanase C (2020). Caveolae as Potential Hijackable Gates in Cell Communication. Front Cell Dev Biol.

[10] Teo JL, Gomez GA, Weeratunga S (2020). Caveolae Control Contractile Tension for Epithelia to Eliminate Tumor Cells. Dev Cell.

[11] Bae GD, Park EY, Kim K (2019). Upregulation of caveolin-1 and its colocalization with cytokine receptors contributes to beta cell apoptosis. Sci Rep.

[12] Martínez M, Martínez NA, Miranda JD (2019). Caveolin-1 Regulates P2Y2 Receptor Signaling during Mechanical Injury in Human 1321N1 Astrocytoma. Biomolecules.

[13] Sanna E, Miotti S, Mazzi M (2007). Binding of nuclear caveolin-1 to promoter elements of growth-associated genes in ovarian carcinoma cells. Exp Cell Res.

[14] Zeng Y, Chen M, Ganesh S (2020). Clinicopathological and prognostic significance of caveolin-1 and ATG4C expression in the epithelial ovarian cancer. PLoS One.

[15] Pisano S, Pierini I, Gu J (2020). Immune (Cell) Derived Exosome Mimetics (IDEM) as a Treatment for Ovarian Cancer. Front Cell Dev Biol.

[16] Wang L, Wu J, Song S (2021). Plasma Exosome-Derived Sentrin SUMO-Specific Protease 1: A Prognostic Biomarker in Patients with Osteosarcoma. Front Oncol.

[17] Peng P, Yan Y, Keng S (2011). Exosomes in the ascites of ovarian cancer patients: origin and effects on anti-tumor immunity. Oncol Rep.

[18] Skryabin GO, Komelkov AV, Galetsky SA (2021). Stomatin is highly expressed in exosomes of different origin and is a promising candidate as an exosomal marker. J Cell Biochem.

[19] Rausch V, Bostrom JR, Park J (2019). The Hippo Pathway Regulates Caveolae Expression and Mediates Flow Response via Caveolae. Curr Biol.

[20] de Souza GM, de Albuquerque Borborema ME, de Lucena TMC (2020). Caveolin-1 (CAV-1) up regulation in metabolic syndrome: all roads leading to the same end. Mol Biol Rep.

[21] Sahay B, Mergia A (2020). The Potential Contribution of Caveolin 1 to HIV Latent Infection. Pathogens.

[22] Ling X, Li Y, Qiu F (2020). Down expression of lnc-BMP1-1 decreases that of Caveolin-1 is associated with the lung cancer susceptibility and cigarette smoking history. Aging (Albany NY).

[23] Aarhus M, Bruland O, Sætran HA (2010). Global gene expression profiling and tissue microarray reveal novel candidate genes and down-regulation of the tumor suppressor gene CAV1 in sporadic vestibular schwannomas. Neurosurgery.

[24] Bélanger MM, Roussel E, Couet J (2004). Caveolin-1 is down-regulated in human lung carcinoma and acts as a candidate tumor suppressor gene. Chest.

[25] Mao X, Tey SK, Ko FCF (2019). C-terminal truncated HBx protein activates caveolin-1/LRP6/β-catenin/FRMD5 axis in promoting hepatocarcinogenesis. Cancer Lett.

[26] Moreno J, Escobedo D, Calhoun C (2021). Arterial Wall Stiffening in Caveolin-1 Deficiency-Induced Pulmonary Artery Hypertension in Mice. Exp Mech.

[27] Vykoukal J, Fahrmann JF, Gregg JR (2020). Caveolin-1-mediated sphingolipid oncometabolism underlies a metabolic vulnerability of prostate cancer. Nat Commun.

[28] Ye JH, Shi JJ, Yin X (2020). Elevated Expression of CAV1 is Associated with Unfavorable Prognosis of Patients with Breast Cancer Who Undergo Surgery and Neoadjuvant Chemotherapy. Cancer Manag Res.

[29] Zhu Q, Zhan D, Zhu P (2020). CircAKT1 acts as a sponge of miR-338-3p to facilitate clear cell renal cell carcinoma progression by up-regulating CAV1. Biochem Biophys Res Commun.

[30] Wang X, Lu B, Dai C (2020). Caveolin-1 Promotes Chemoresistance of Gastric Cancer Cells to Cisplatin by Activating WNT/β-Catenin Pathway. Front Oncol.

[31] Shi YB, Li J, Lai XN (2020). Multifaceted Roles of Caveolin-1 in Lung Cancer: A New Investigation Focused on Tumor Occurrence, Development and Therapy. Cancers (Basel).

[32] Sayhan S, Diniz G, Karadeniz T (2015). Expression of caveolin-1 in peritumoral stroma is associated with histological grade in ovarian serous tumors. Ginekol Pol.

[33] Liu B, Zhang J, Yang D (2019). miR-96-5p promotes the proliferation and migration of ovarian cancer cells by suppressing Caveolae1. J Ovarian Res.

[34] Davidson B, Nesland JM, Goldberg I (2001). Caveolin-1 expression in advanced-stage ovarian carcinoma - a clinicopathologic study. Gynecol Oncol.

